# Effects of a nurse-led family education intervention on the daily living skills of children with autism

**DOI:** 10.3389/fpsyt.2025.1706047

**Published:** 2025-12-09

**Authors:** Dandan Ding, Hiping Xu, Xiaoyan Du, Xiangdan Su, Lijie Huang, Xueqin Zhang, Yawen Hu, Zhaunli Wang, Xuehan Li, Jingjing Dai, Yiru Zhu

**Affiliations:** Department of Child Development and Behavior, The Third affiliated Hospital of Zhengzhou University, Zhengzhou, China

**Keywords:** autism, daily living skills, family education, intervention, nurse-led

## Abstract

**Background:**

Patients with autism spectrum disorders have different degrees of daily living skills defects, which can negatively impact their ability to integrate into society. However, the provisioning of nurse-led family education interventions is often not prioritized, and whether family interventions for the parents of children with autism can impact the daily living skills of those children remains poorly understood.

**Objective:**

To investigate the effects of a nurse-led family education intervention on the daily living skills of children with autism.

**Methods:**

Convenience sampling was used to select children with autism who were hospitalized in the Department of Child Development and Behaviour at a tertiary grade hospital in Zhengzhou, Henan Province, during the period from June 2024 to March 2025. To avoid cross-group contamination, hospitalized patients in the second ward of the Department of Child Developmental Behaviour were used as the intervention group, and hospitalized patients in the first ward of the Department of Child Developmental Behaviour were used as the control group. Researchers collected data before, directly after, and 3 months after the intervention using unified guiding language.

**Results:**

Compared with those before the intervention, directly after intervention and those 3 months after the intervention, the Barthel Index (BI) and Instrumental Activities of Daily Living Scale (IADL) scores for the intervention group significantly increased, whereas the ABC score for that group significantly decreased (*P* < 0.05). Compared with those of the control group, the intervention group had significantly higher BI and IADL scores and significantly lower ABC scores (*P* < 0.05).

**Conclusions:**

Nurse-led family education interventions can improve the daily living skills of children with autism, improve their behavioural characteristics, and promote their effective integration into social life.

## 1 Introduction

Autism spectrum disorders (ASDs) are a group of neurodevelopmental disorders that are primarily characterized by social communication difficulties; communication impairments; and restricted, repetitive, and stereotyped behaviours (39). Recent data from the U.S. Centers for Disease Control and Prevention (CDC) show that the incidence of autism has increased from 1 in 45 to 1 in 36 (1, 2). This increasing incidence highlights the urgent need for effective interventions to improve the quality of life of children with ASD and alleviate the burden both on their families and society.

Despite poor daily living skills (DLSs) not being considered core symptoms of ASD, individuals with autism spectrum disorder exhibit significant deficits in these skills because of language communication barriers and a lack of social skills (3). Such individuals often struggle to complete self-care tasks, such as dressing, personal hygiene, and eating in their daily lives. Studies have shown that limitations to daily living skills prevent individuals with autism from living independently, which causes them to require long-term family care, thereby imposing a heavy economic burden on their families and society (4, 5).

Daily living skills (DLSs) consist of three aspects: personal skills (self-care), domestic skills (home maintenance), and community skills (school/community life) (6). DLSs are multilayered, whereas activities of daily living (ADLs) refer to more basic life functions (e.g., brushing teeth, dressing, and personal hygiene), which directly impact quality of life. The higher-level instrumental activities of daily living (IADLs) include more complex domestic and community tasks (e.g., financial management, meal preparation, and laundry), which directly influence an individual’s ability to function independently in their environment (7). The acquisition of these skills affects one’s capacity for self-sufficiency and independent living in daily settings. Studies have shown that higher DLS proficiency in individuals with ASD is correlated with better engagement in future learning, daily life, and employment (8).

Studies have shown that individuals with ASD have limited daily living skills (DLS), with approximately 88% unable to live independently in adulthood and approximately 60% still requiring care (9). Even among high-functioning individuals with ASD, only 32% can achieve independent living. DLS refers to an individual’s ability to manage their self-care and daily tasks, and it represents the most basic life skills needed for independent living (10). Mastering such skills is particularly crucial for individuals with ASD, as improving DLS proficiency enhances quality of life and facilitates social integration. Therefore, DLS training should serve as a key focus and goal in treatment plans for individuals with autism spectrum disorder.

The family-centered intervention model for autism designates the family, instead of the child, as the core of the intervention. Through systematic training and parental empowerment, daily life scenarios are converted into opportunities to foster the development of children’s communication, social, and behavioral skills, thus attaining personalized and sustainable intervention. Research has indicated that family-centered remote support may substantially enhance the prognosis and quality of life for individuals with autism spectrum disorder and their families (11). The research findings demonstrate that the stepped-parenting class intervention model can enhance the social and self-care capabilities of children with autism spectrum disorder (12).

The provisioning of family education interventions has emerged as a promising approach to enhancing the DLS of children with ASD (13). Parents and caregivers play a central role in the daily lives of these children, and providing such children with appropriate knowledge and skills can potentially translate into improved outcomes (14). Nurses, with their expertise in patient education, health promotion, and behavioural management, are well positioned to lead such family-centred interventions. However, the literature on nurse-led family education interventions for improving DLS in children with ASD remains scarce. Most studies are focused on behavioural therapy or pharmacological interventions, leaving a gap in our understanding of the effectiveness of nurse-led, family-based educational strategies.

Nurses have professional medical knowledge, nursing skills and good communication skills, and they can play a key role in family education interventions. The program is aimed at helping children with autism improve their daily living skills, including such areas as self-care, social interaction and cognitive understanding, through nurse-led interventions while enhancing the capacity for family care, improving the family intervention environment and promoting children’s overall development.

## 2 Methods

### 2.1 Design and participants

Convenience sampling was used to select children with autism who were hospitalized in the Department of Child Development and Behaviour at a tertiary grade hospital in Zhengzhou, Henan Province, during the period from June 2024 to March 2025. The inclusion criteria were as follows: (1) qualified subjects met the diagnostic criteria of autism spectrum disorder (autism) in the expert consensus on family intervention for young Chinese children with autism spectrum disorder (15); (2) qualified subjects were ≥2 years old; (3) qualified subjects had received no other interventions prior to enrolment; and (4) the family members of qualified subjects agreed to participate in the study and signed the consent form. The exclusion criteria were as follows: (1) children with schizophrenia did not qualify; (2) growth retardation caused by other inducements was disqualifying; and (3) severe organ dysfunction was disqualifying. Sample size calculation is based on the mean of two samples formula (16)n1=n2 = 2[(μα+μβ)σ/δ]^2^, The inspection level is set to α=0.05、β=0.10; According to the previous literature data with σ=0.85 and δ=1.05, the sample size for a single group is calculated to be 32 cases and considering a sample loss rate of 20%, the total number of included samples was 76, comprising 38 cases in the intervention group and 38 cases in the control group. Using the coin toss method, divide the two wards of the Department of Child Developmental Behavior into two groups, with one ward as the intervention group and the other ward as the control group, to avoid contamination. All parents of the selected children voluntarily participated and signed informed consent forms. This study was reviewed by the hospital’s medical ethics committee (2022–391).

### 2.2 Measurement

#### 2.2.1 Demographic and clinical characteristics

Basic information on the child included sex, age, place of residence and the child’s medical history, Check for the presence of comorbidities and the severity of diseases in the medical information system. Basic Information on the parents included age, educational background, occupation type, family economic status, etc.

#### 2.2.2 Assessment of the basic activities of daily living

The Barthel Index (BI) is used to evaluate a child’s 10 basic self-care abilities, which include eating, grooming, washing, bathing, toileting, dressing, transfer, walking/wheelchair use, stair climbing, and bowel/bladder control (17, 18). Each item is scored using a 4-point Likert scale (0/5/10/15 points) based on the child’s degree of dependence on others. A higher score indicates a stronger level of independence and better self-care ability regarding basic daily activities. The Chinese version of the BI has good reliability and validity (Cronbach’s α coefficient >0.8) and is widely used for baseline assessments of self-care ability (19).The Barthel Index has been used to evaluate the basic activities of daily living of autism (20), and the Cronbach’s α coefficient of the scale in this study was 0.738.

#### 2.2.3 Instrumental activities of daily living assessment

The Instrumental Activities of Daily Living Scale (IADL) is adopted to evaluate the complex social functions, including shopping, cooking, financial management, the use of medication, and the use of transportation, of children with autism (21). These activities reflect a child’s higher level of adaptive and independent living ability within family and social environments. This scale consists of 8 items, with each item scored as a 1 or 0 on the basis of ability, for a total score of 0–8 points. The higher the score is, the better the self-care ability. The Chinese version of the IADL has undergone rigorous revision and validation processes, and it exhibits good reliability and validity (22); it can be used to effectively assess the developmental level of children with autism in regard to these complex life skills and to accurately identify their strengths and weaknesses in social adaptation (23).The IADL has been utilized in patients with autism (24). In the present study, the Cronbach’s α coefficient was determined to be 0.710.

#### 2.2.4 Autism behaviour scale

The ABC scale was developed by Krug, and the Chinese version of the scale was revised by Professor Yang (25) and consists of 57 items covering five dimensions: sensation, communication, physical movement, language, and self-care. The scale uses a 4-level Likert scoring method, with each item assigned different scores (1–4 points) on the basis of its importance on the scale. The assessment is conducted by parents or individuals who have lived with the child for more than 2 weeks. The Chinese version of the ABC scale has good reliability and validity and provides a basis for comprehensively understanding children’s behavioural characteristics and developmental statuses to -inform family education interventions (26), and the Cronbach’s α coefficient of the scale in this study was 0.806.

### 2.3 Intervention

#### 2.3.1 Formation of the intervention team

The research team in this study comprises six members, including one head nurse, two pediatric developmental behavior specialist nurses, one nursing postgraduate student, one pediatric developmental behavior physician, and one rehabilitation therapist. Team members will undergo unified training to comprehend the family education intervention plan and its details. After completing the training and passing the assessment, the nurses will be tasked with implementing the intervention plan step-by-step. The head nurse will conduct regular supervision and guidance, verify the execution of the intervention measures, and monitor the feedback from the family members.

#### 2.3.2 Formulate the intervention plan

In the initial stage, the components and steps of the intervention plan were developed via a literature review and expert consultation. Domestic and foreign databases such as PubMed, the Cochrane Library, CINAHL, Web of Science, CNKI, Wanfang, etc., were searched, and the relevant literature published over the previous 10 years was reviewed. Moreover, the researchers participated in daily living skills training courses and initially constructed a content framework for a daily living skills intervention for children with autism. The daily living skills family education intervention for children with autism, which is led by nurses, includes six components: (1) basic ASD knowledge and understanding; (2) skill assessment and goal setting; (3) basic self-care skills training; (4) home and community adaptation skills; (5) practical application of commonly used training methods; and (6) family training plans and implementation. A group of experts were invited to review the nurse-led intervention draft, and child development behaviour doctors, nursing educators, special education experts for children with autism, and clinical nurses all examined the feasibility and applicability of the plan. The expert feedback indicated that the content of the plan was feasible. The steps and components of the intervention plan are shown in **Table 1**.

**Table 1:** Daily living skills family education intervention program.

Intervention module	Content	Family goals	Intervention forms	Interveners
1.Basic ASD knowledge and understanding (parental empowerment)	The core characteristics of ASD: social communication differences (eye contact, facial expressions, nonverbal interactions); repetitive behaviour and special interests; and abnormal perception (sensitive/sluggish) The Development Law of Daily Living Skills	Parents can accurately describe their children’s three core characteristics and analyse how these characteristics affect daily living skills learning. Enable parents to clearly understand ASD and comprehend the developmental characteristics of their children’s life skills, laying a cognitive foundation for subsequent interventions.	Special lectures and distribution of materials.	Nurse
2. Skill assessment and goal setting (scientific planning)	Skill baseline assessment: Use of the life skills checklist to evaluate self-care (dressing/eating/washing/toiletry), home skills (organizing items/simple household chores), and community adaptation (shopping/traffic rules). Target priorities of the sorting selection criteria: safety and health related (such as toileting); independence needs (such as eating by oneself), and family pain points (such as reducing morning procrastination). Task decomposition technique: breakdown complex skills into 5–8 actionable steps (such as “brushing teeth” → holding toothbrush→ squeezing toothpaste, etc.).	Parents complete their child’s current skill assessment form and mark items as having been independently completed, required assistance, or unable to complete. Parents select 1–2 priority goals (e.g. independently putting on and taking off jackets and eating with a spoon) and clarify the intervention sequence. Parents breakdown the target skills into specific steps and create a step-by-step diagram (including text and images).	Special lectures and one-on-one guidance.	Doctor、Nurse
3. Basic self-care skills training (core module)	Eating skills: proper use of utensils (spoon → fork → chopsticks), cleaning of plates (delivering bowls to sink), and food selection (using picture communication board). Dressing skills: - identifying the front and back of clothing and the order of putting on and taking off clothes (take off first, put on later). Handle buttons/zippers (use of auxiliary tools) Washing skills: six step hand washing method (visual flowchart), brushing timing (hourglass/timer), combing hair/wiping face. Toilet skills: - expression of toilet needs (pictures/gestures), taking off pants → sitting on the toilet → cleaning → flushing → hand washing process.	Within 4 weeks: The child independently uses a spoon to finish half a bowl of rice (spills less than 10%) and then sends the bowl to the kitchen after meals. Within 6 weeks: The child independently completes the process of putting on and taking off a pullover (including adjusting the collar) with an accuracy rate of ≥ 80%. Within 4 weeks: The child independently completes the entire process according to the hand washing flowchart and proactively uses a towel to dry without verbal prompts. Within 8 weeks: the number of accidents during daytime toileting are reduced by 50%, and the child actively points to pictures of the restroom to express needs.	Scenario simulation teaching, decomposition step demonstration (such as dressing step decomposition demonstration), family practical practice (parental guidance), visual support, chain method, and reinforcement system.	Nurse
4. Home and community adaptation skills (generalized application)	Home responsibilities: returning items (toys → boxes), simple household chores (cleaning tables and watering flowers), safe behaviour (power switch rules), making phone calls (dialling, communication content, and ending calls). Community participation: Financial management: understanding coins and a simple shopping budget; supermarket shopping, including picking up goods → queueing → paying (simulation game); using public transportation: ride/seat recognition/grab handrails; and public etiquette (quiet gestures) Security skills: Remembering home address/phone number (visual card), identifying dangerous places (picture teaching), and refusing strangers (scenario exercise).	The child classifies and stores their toys before going to bed every day, with an accuracy rate of ≥ 90%. Accompanied, the child can independently pick up 3 items from the list at the supermarket and place them at the checkout counter. Children can present their family contact card to trusted uniformed personnel (police/security).	On site scenario teaching. Family task assignment and guidance.	Nurse
5. Practical application of commonly used training methods (parental skills)	Visual support: Create personalized schedules (morning/bedtime process) and use “first then” cards (dress first → go out to play later) Structured teaching: breakdown skills into small tasks and teach according to a fixed process, such as the structured process of dressing. Scenario simulation: role-playing (shopping/riding) and video demonstration (playing videos of peers brushing their teeth) Chain lock method: Backwards chain lock: parents complete the first N steps, children complete the last step (such as only zip up when dressing), and there is a gradually move towards independence.	Parents should create a morning flowchart for their children and use it continuously for 7 days to record any changes to their child’s cooperation level; choose 1–3 skills to decompose and teach according to the process; select one target skill and practice it three times a day using the backwards chain locking method, recording the independent situation for each step; and simulate community scenarios (such as practising payment using a toy checkout counter) twice a week and record the process for review.	Method explanation and demonstration case analysis Simulation training (parent practice method, teacher guidance)	rehabilitation therapist
6. Family training plan and implementation(sustainability)	Plan formulation: Short term: Weekly plan (specifying daily training skills/frequency/reinforcement); Long term: Monthly goal (such as achieving independent toileting within 3 months). Data recording: Use the Skill Progress Table to record the number of independent completion times, the required prompt level (limb → gesture → visual → independent), and the frequency of problematic behaviours. Environmental adjustments: reduce interference (turn off TV during training), establish skill corners (paste process diagrams in the washing area), and ensure sensory friendliness (choose non fluorescent lighting).	Provide families with systematic and sustainable training programs to ensure the sustained and effective implementation of life skills interventions.	Panel Sessions. One on one customized solution. Tracking guidance (regularly understanding the implementation status of the plan, adjusting and optimizing it).	Nurse

#### 2.3.3 Implementation plan

On the basis of routine health education, the intervention group received a nurse-led daily living skills family education intervention program. The intervention was gradually conducted in accordance with the project plan. The intervention group was provided with a three-month health education program. The average duration of hospital stay for patients in the department is six weeks. Therefore, a six-week group meeting was organized during hospitalization, and a six-week telephone follow-up was conducted after discharge. The intervention content of six modules will be presented during six group meetings. Subsequent to each group meeting, tasks relevant to the module content will be assigned, and notifications will be issued to prompt group check - ins. Ultimately, the completion status of these tasks will be tallied. the group meeting utilized one theme per week, and after a brief introduction or summary, the group focused on the theme of the week. During the intervention period, methods such as health education, peer interaction, and sharing experiences were adopted. At the same time, a WeChat group was established to remind parents every week and to clock in per the requirements. To avoid participation fatigue, face-to-face interventions had a duration of for 40–60 minutes each.

The control group received routine health education. The control group was subjected to routine health education, encompassing disease knowledge dissemination, rehabilitation training method instruction, dietary guidance, and daily life skills training guidance, among other aspects. Three months later, the intervention content was delivered to the control group, accompanied by guidance and responses to inquiries. Participants in the control group were asked to follow the official WeChat account (317 nurses) created by the researchers, and they were showed how to find historical information and view popular science articles. Health education-related materials were sent to participants in the form of text, pictures or videos through official WeChat accounts every week. In addition, weekly telephone follow-up was conducted with participants to assess their understanding of the health education content and address any participant questions.

### 2.4 Data collection

The primary outcomes were assessments of daily living skills proficiency, which were evaluated using the Barthel Index (BI) and the Instrumental Activities of Daily Living (IADL) scale. The secondary outcomes included the measurement of the behavioural characteristics and developmental status of children using the Autism Behaviour Scale (the ABC Scale). Researchers, under standardized instructions, underwent unified training and used standardized instructions to conduct measurements prior to the intervention, after the intervention, and during the 3-month follow-up. Additionally, general information was collected at baseline, and weekly records for program participation and satisfaction with the intervention protocol were maintained during the intervention period.

All the researchers were master’s degree-registered nurses with professional training in daily living skills intervention. The intervention protocol was evidence-based and had its feasibility confirmed through a pretest pilot study. During the intervention, researchers established positive relationships with the participants to reduce the dropout rates across groups. In the follow-up phase, the researchers contacted the participants via phone or WeChat, guiding their task monitoring through WeChat while providing encouragement and support.

### 2.5 Data analysis

We used IBM SPSS 22.0 for data analysis. Statistical descriptions are summarized as either the mean and standard deviation or frequency and percentage, depending on the type of variable. For statistical inference, the chi‐square test was used to compare the categorical variables between groups, when appropriate. Two independent samples *t* tests and paired *t* tests were used for comparisons between two groups or within groups at different time points. Repeated measures analysis of variance (ANOVA) was applied to assess the change trend in the scale scores across the three time points. *p* < 0.05 was considered to indicate statistical significance.

## 3 Results

### 3.1 Participant characteristics

Among the 76 parents of children with autism who participated in this study, 38 were placed in the intervention group and 38 in the control group. The satisfaction rate with the health education intervention program of the parents of sampled children reached 100%. There were 38 cases in the intervention group (32 males and 6 females); the ages ranged from 2 to 5 years, with an average of 4.82 ± 1.48 years; and the average duration of illness was 2.47 ± 0.66 years; Comorbidities (17 cases; 21 cases without); Severity of the disease (mild 22 cases, moderate 10 cases, severe 6 cases). The 38 patients in the control group included 25 males and 13 females; their ages ranged from 2 to 5 years, with an average of 4.83 ± 1.94 years; and the average duration of illness was 2.51 ± 0.67 years; Comorbidities (13 cases; 25 cases without); Severity of the disease (mild 16 cases, moderate 14 cases, severe 8 cases). There was no statistically significant difference in general information between the two groups of patients (*P*>0.05).The general demographic data of the sampled parents were compared separately, and no statistically significant difference was found between the two groups (*P*>0.05), indicating that the two groups of sampled parents had good balance and comparability in the comparison of general demographic data (See **Table 2**).

**Table 2:** Comparison of parents’ general information between the intervention group and the control group both before and after the intervention.

Variable	Intervention group [*n* = 38, *n* (%)/( ± *S*)]	Control group [*n* = 38, *n* (%)/(± *S*)]	*t*/*X*^2^	*P*
Age (years)	37.23 ± 10.12	35.28 ± 7.36	0.959	0.341
Sex			0.736	0.368
female	32(84.21%)	34(89.47%)		
male	6(15.79%)	4(10.53%)		
Education			2.554	0.279
High school or below	18(47.37%)	16(42.11%)		
vocational education	8(21.05%)	14(36.84%)		
bachelor’s degree or above	12(31.58%)	8(21.05%)		
Monthly household income			0.332	0.847
<5000	19(50.00%)	21(55.26%)		
5000~10000	16(42.11%)	15(39.48%)		
>10000	3(7.89%)	2(5.26%)		
Career			0.512	0.744
full-time job	17(44.74%)	19(50.00%)		
part-time job	5(13.15%)	6(15.79%)		
unemployed	16(42.11%)	13(34.21%)		
Place of residence			0.644	0.322
town	20(52.63%)	23(60.53%)		
rural area	18(47.37%)	15(39.47%)		

### 3.2 Between groups comparison of daily living skills

There was no statistically significant difference in the total BI or IADL scores between the two groups of sampled before the intervention (*P*>0.05). After the intervention, the total BI and IADL scores in the intervention group were significantly greater than those in the control group (*P* < 0.05). Repeated measures analysis of variance was conducted on the BI and IADL total scores for the research subjects at different times. The intergroup factor was grouping (control group and intervention group), the intragroup factor was time (time before intervention, directly after intervention, and 3 months after intervention), and the interaction was grouping x time. The results reveal a statistically significant difference in the total BI and IADL scores between the intervention group and the control group (*P* < 0.01), and there were statistically significant differences in the total BI and IADL scores at different times (*P* < 0.05). These results indicate that health education may have a positive influence on DLS following the intervention. See **Table 3** and **Table 4**.

**Table 3:** Comparison of the BI scores between the two groups.

Group	BI score( ± *S*)				
	T0	T1	T2	Ftime	*P*
Intervention group	73.42 ± 13.66	82.42 ± 18.95	91.05 ± 13.81	13.341	0.000
Control group	72.89 ± 17.69	73.47 ± 18.40	80.67 ± 14.35	2.862	0.067
T	0.145	2.088	3.191	Fbetween group=7.739	Fbinter action=2.265
*P*	0.885	0.040	0.002	0.007	0.107

Fbetween group=7.739, *P* < 0.05, partial η² = 0.095; Finter action=2.265, *P* < 0.05, partial η² = 0.030.

**Table 4:** Comparison of the IADL scores between the two groups.

Group	IADL score( ± *S*)				
	T0	T1	T2	Ftime	*P*
Intervention group	0.86 ± 1.45	2.94 ± 1.37	4.50 ± 0.72	103.35	0.000
Control group	0.89 ± 1.06	1.07 ± 1.47	1.86 ± 1.10	7.434	0.001
T	-0.090	5.708	12.202	Fbetween group=67.083	Fbinter action=61.299
P	0.928	0.000	0.000	0.000	0.000

Fbetween group=67.083, P < 0.01, partial η² = 0.475; Finter action=61.299, P < 0.01, partial η² = 0.453.

**Figure 1:**
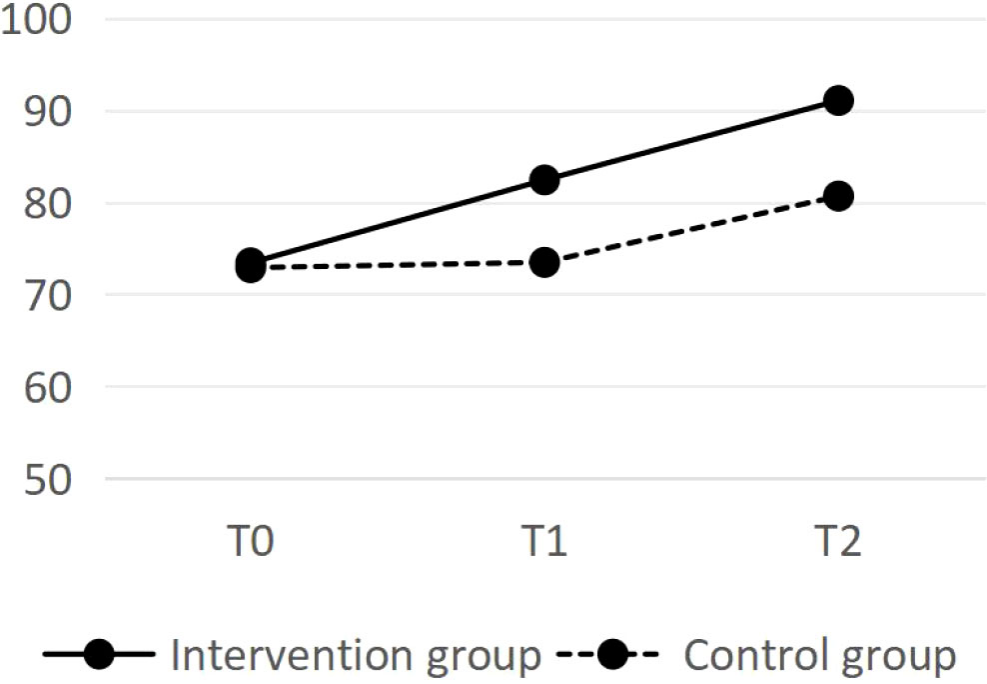
The changes in BI scores.

**Figure 2:**
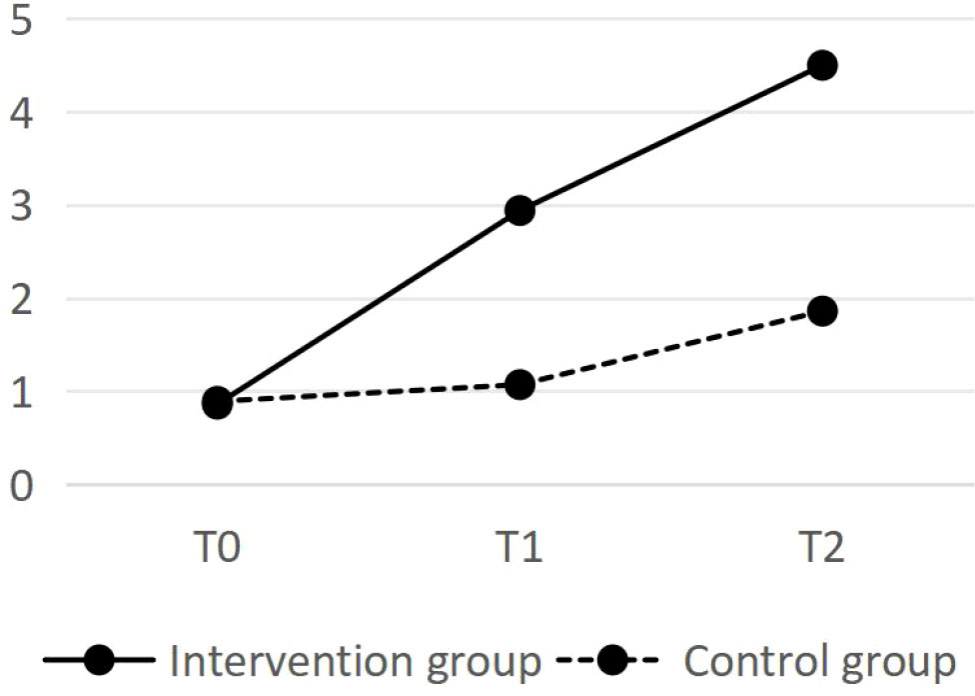
The changes in IADL scores.

The changes in BI scores and IADL scores for the two groups are shown in **Figures 1** and **2**, respectively. The BI score and IADL score of the intervention group significantly increased throughout the intervention, and the trend change in the scores during the follow-up period was also significant. After the intervention, the scores of the control group remained essentially unchanged, whereas a slight increase was observed during the 3-month follow-up.

### 3.3 Comparison between groups for ABC

There was no statistically significant difference found in the total ABC score between the two groups of sampled before the intervention (*P*>0.05). After the intervention, the total ABC score of the intervention group was significantly lower than that of the control group (*P* < 0.05). Repeated measures analysis of variance was conducted on the total ABC scores at various time points, and the results revealed a statistically significant difference in the total ABC scores of the intervention group compared with those of the control group (*P* < 0.05). The differences in the ABC total scores at different times were statistically significant (*P* < 0.05). An interaction effect was found between the total ABC score and time (*P* < 0.05), indicating that the intervention group and the control group followed different trends in the changes regarding the total ABC score at different time points. These results indicate that health education may exert a positive influence on core behavioural symptoms after intervention. See **Table 5**.

**Table 5:** Comparison of the ABC scores between the two groups.

Group	ABC score( ± *S*)				
	T0	T1	T2	Ftime	P
Intervention group	108.13 ± 26.83	82.81 ± 29.76	71.81 ± 25.31	32.45	0.000
Control group	107.47 ± 29.27	97.28 ± 20.35	89.75 ± 27.73	6.817	0.002
T	0.102	-2.474	-2.927	Fbetween group=4.930	Finter action=4.258
P	0.919	0.016	0.005	0.029	0.016

Fbetween group=4.930, *P* < 0.05, partial η² = 0.062; Finter action=4.258, *P* < 0.05, partial η² = 0.054.

**Figure 3:**
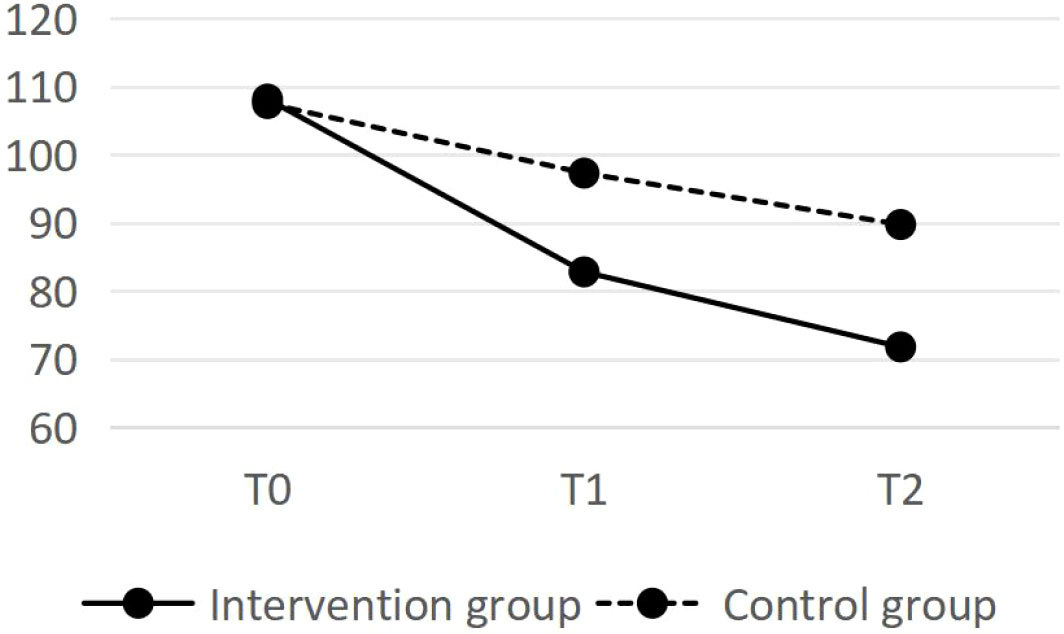
The changes in ABC scores.

The changes in the ABC scores for the two groups are shown in **Figure 3**. The ABC score of the intervention group significantly decreased as the intervention progressed. After the intervention, the scores of the control group remained essentially unchanged, with a slight decrease observed during the 3-month follow-up.

## 4 Discussion

The results of this study confirm that nurse-led family health education has a significant and sustained effect on improving the daily living skills and behavioural characteristics of children with autism. After intervention and 3-month follow-up, the Basel Index (BI) and Instrumental Activities of Daily Living (IADL) scores of the children in the intervention group significantly increased, whereas the Autism Behaviour Scale (ABC) scores significantly decreased (*P* < 0.05), indicating that this intervention model has the capacity to not only effectively promote the improvement of children’s basic self-care abilities (such as eating, dressing, and washing) and instrumental daily activity abilities (such as simple household chores and shopping assistance) but also to improve their core behavioural symptoms, thereby laying a foundation for effective integration into family and society.

Research has shown that the level of daily living skills is an important factor in predicting whether a child will have the capacity to live independently in the future (27). A systematic review of 15 studies on parental-mediated DLS interventions for children with ASD revealed that parent training programs can effectively improve the daily living skills of children with autism (28). In the literature on improving the daily living skills of children with autism, a daily living skills intervention was conducted for parents of children with autism (13). After the intervention, the daily living skills of the intervention group were significantly greater than those of the control group were. Gong also reached a similar conclusion following an intervention with parents of children with autism (12). The results of the current study are consistent with those of previous research. In other words, family intervention can strengthen the adaptive behaviour training of children, help them breakdown their daily living skills (such as brushing teeth and tying shoelaces) into operable steps, and teach training techniques to their parents via video demonstrations, physical operations, and other methods to promote these children’s acquisition of daily living skills (29). The reason for this may be that one of the core obstacles for children with autism is their lack of social communication and daily living skills, and within the principle living environment for children, the effectiveness of educational interventions directly affects their functional improvement (30). Children with autism are more sensitive to environmental changes, and the familiarity of scenes in their family environments can reduce their anxiety and promote the natural transfer of skills (31). These results are consistent with the findings of early family care interventions (32); training in the family environment has stronger situational consistency, and such children are more likely to apply skills to practical life.

Repeated-measures ANOVA reveals that the daily living skills scores of the children in the intervention group tended to differ from those of the children in the control group at different time points. The intervention group remained above baseline for 3 months after completion, indicating that nurse-led family health education exerts a sustained effect. This finding is consistent with research conclusions concerning the long-term impact of family intervention on the maintenance of daily living skills in children with autism (33). The results indicate that the cultivation of daily living skills in children with autism is a long-term process that requires persistence (34). Future research should extend the follow-up period and provide regular follow-up guidance to help parents continuously optimize their training strategies, ensure the long-term effectiveness of intervention, and reduce the burden of family care (35). Family education plays a central role in the rehabilitation process of children with autism. As the main executor of such education, parents can dynamically adjust intervention strategies on the basis of the child’s daily routine, interests, preferences, and behavioural characteristics (36). Parents can provide training to their children at any time and any place throughout their daily lives, integrating training into their daily lives and normalizing training. Continuous emotional interactions within the family environment, such as the encouragement of language and physical contact, can increase children’s motivation to participate. Research has shown that children in the intervention group show a greater degree of cooperation in family training, which is consistent with the conclusion of a study on the effect of family comprehensive nursing in children with autism, which found that emotional support is a key factor in improving intervention compliance.

The significant decrease in ABC scale scores (which indicates a reduction in behavioural problems) further supports the effectiveness of the intervention (37). Children with autism often display problems such as stereotyped behaviour and emotional instability, which not only affects their acquisition of daily living skills but also may hinder their social integration. In nurse-led family health education, by guiding parents in the identification of their children’s behavioural triggers and in the use of positive reinforcement (such as reward mechanisms) and behaviour correction techniques (such as alternative behaviour training), parents are guided in intervening in their children’s bad behaviour in a timely manner within the family setting, which can gradually reduce stereotyped behaviour and emotional problems. Moreover, nurses can dynamically adjust intervention strategies by recording project participation on a weekly basis, thereby ensuring that parents can master effective behaviour management methods and achieve continuous improvement in their children’s ABC scores.

Nurses, as professional medical personnel, play an irreplaceable leading role in family education interventions. Nurses possess solid medical knowledge and nursing skills and have the capacity to accurately assess the condition and developmental stages of paediatric patients from a professional perspective, thereby providing a basis for the development of personalized intervention plans (38). Moreover, systematic health education for parents, including skill operation guidance, behaviour guidance strategies, etc., should be provided to empower parents to act as the executors of intervention. This model makes up for the limitations of traditional routine health education by providing continuous interaction during hospitalization and enabling practical extension in family settings, thereby assisting children in repeatedly practicing skills in a familiar environment, forming stable behavioural habits, and achieving continuous improvement in their BI and IADL scores. During the follow-up process, any problems that the child may have encountered during the training process should be promptly identified, and targeted guidance and adjustments should be provided. Training methods or rhythms can be adjusted on the basis of the child’s characteristics to ensure the effectiveness of the intervention. In addition, nurses can provide psychological support to parents, helping them to alleviate negative emotions such as anxiety and stress that result from taking care of children with autism, thereby enhancing their confidence and enthusiasm for intervention and ensuring the smooth implementation of intervention plans.

This study takes nurses as the intervention leader, which is aligned with the patient-centred concept and fully leverages the professional advantages of nurses in chronic disease management and family health guidance. Compared with the traditional intervention model, which is led by doctors or rehabilitation therapists, nurse-led family health education places more emphasis on family participation. By reducing the difficulty of parents’ operations and improving the accessibility of intervention, parents’ enthusiasm for participation can be enhanced. The high level of satisfaction that was noted during the intervention period for this study also confirms this point.

### 4.1 Research limitations

In this study, a convenience sampling method was used, and the singularity of the sample area and case source could affect the extrapolation of the results. In the future, multicentre and large sample studies need to be conducted for further verification. Although a 3-month follow-up was included following the intervention, the recovery of children with autism is a long-term process, and the follow-up time should be extended in the future (such as 6 months or 1 year) to observe the longer-term stability of the intervention effect.

## 5 Conclusion

The nurse-led health education intervention implemented in this study effectively improved the daily living skills of children with autism and provided practical guidance for family rehabilitation. In the future, such intervention plans can be further optimized, such as through a combination of information technology and the introduction of multidisciplinary teams to increase the accuracy and enjoyability of interventions. Moreover, strengthening psychological support for parents, alleviating their caregiving pressure, improving the sustainability and compliance of interventions, and ultimately helping children with autism achieve higher-quality social integration are necessary.

## Data Availability

The raw data supporting the conclusions of this article will be made available by the authors, without undue reservation.
